# HPV Genotypes distribution in Indian women with and without cervical carcinoma: Implication for HPV vaccination program in Odisha, Eastern India

**DOI:** 10.1186/s12879-016-2136-4

**Published:** 2017-01-05

**Authors:** Rashmirani Senapati, Bhagyalaxmi Nayak, Shantanu Kumar Kar, Bhagirathi Dwibedi

**Affiliations:** 1 Viral Research and Diagnostic Laboratory, Regional Medical research Centre (ICMR), Nalcosquare, Chandrasekharpur, Bhubanewar, 751023 Odisha India; 2 Department of Gynecologic oncology, Acharya Harihara Regional Cancer Centre, Cuttack, Odisha India; 3 Institute of Medical Science and Sum Hospital, Shiksha O Anusandhana University, Bhubanewar, Odisha India

**Keywords:** HPV genotype distribution, Cervical cancer, Impact of HPV vaccine, Odisha, Eastern India

## Abstract

**Background:**

Considering the limited cross protection offered by the current HPV vaccines, understanding the HPV genotype distribution among the different population is essential in predicting the efficacy of current vaccine and devising new vaccine strategy. The present work aimed at investigating the HPV genotypes distribution among women with and without cervical carcinoma in Odisha, Eastern India.

**Methods:**

A total of 607 participants have been enrolled between January 2014 and June 2016. L1-PCR, sequencing, and E6/E7 nested multiplex type- specific PCR were performed for HPV detection and genotyping. Cytological distribution of 440 cases includes invasive cervical carcinoma or ICC (*n* = 210), inflammatory smear (*n* = 162), normal cytology (*n* = 68). Statistical analyses were performed by using SPSS version 20.0 software and MediCal version 14.10.2(7). A *p*-value of ≤ 0.05 was considered statistically significant.

**Results:**

The overall prevalence of HPV infection was (359/595) 60.33%. Prevalence of HPV infection was 93.80% (197/210) in invasive cervical cancer (ICC) cases, 54.32% (88/162) in inflammatory smear and 19.11% (13/68) in normal cervical cytology. The most prevalent genotype was HPV16 (87.28%) followed by HPV18 (24.56%) and HPV 51(3.46%). The overall prevalence of single type was 76.58% and highest (78.9%) among ICC cases. The most frequent genotype combination after HPV16 + 18(9.4%) was HPV16 + 66 + 68(2.7%) which was frequently observed in inflammatory cytology. Age > 45years, parity ≥3, low socio-economic condition, rural residential area and post menopause state were significantly associated with HPV infection. Multiple infections did not have a significant association with any of the clinicopathological variables (stage, LN metastasis, cell type) except tumor size ≥ 2cm in ICC cases. The impact of 2v, 4v, and 9v vaccines in preventing cervical cancer in Odisha were 89.99, 91.65, and 92.16% respectively.

**Conclusion:**

This data would help planning an appropriate strategy for disease monitoring and provides baseline data for post-vaccination surveillance in the region. The nonavalent vaccine would be significant in preventing cervical carcinoma in Odisha. Hence an effective vaccination program based on regional HPV epidemiological profile along with the cervical cancer screening is necessary to reduce the cervical cancer burden in India.

**Electronic supplementary material:**

The online version of this article (doi:10.1186/s12879-016-2136-4) contains supplementary material, which is available to authorized users.

## Background

Cervical cancer is the fourth most common cancer in women and seventh most common cancer among all the known group of cancers found worldwide [1]. 528,000 new cases of cervical cancer recorded in the year 2012 [1]. Death due to cervical cancer estimated to be 266,000 worldwide. It accounts for 7.5% of all female cancer deaths [1]. 123,000 new cases of cervical cancer with 67,000 deaths recorded in India in the year 2012 [1].

HPV infection plays a central role in causing cervical cancer. Among 184 different HPV genotypes, only 40 diverse types can infect anogenital region which can be classified into 3 classes based on their oncogenic potential. HPV16, 18, 31, 33, 35, 39, 45, 51, 52, 56, 58, 59, 68, 73 and 82 are included in high-risk group while HPV6, 11, 40, 42, 43, 44, 54, 61, 70, 72 and 81 are included in low-risk group whereas HPV 26, 53 and 66 belong to the group of intermediate risk [2, 3]. Considering the limited cross protection offered by the current vaccines, understanding the genotype distribution among different population is essential in predicting the efficacy of current vaccine and devising new vaccine strategy [4]. Epidemiology of HPV infection and pattern of HPV genotype distribution are not well documented in the entire Indian subcontinent which limits the implementation of cervical cancer prevention programs**.** Available reports from studies covering few parts of India show a wide variation in the prevalence of HPV infection and genotypes distribution which is attributed to diversified socio-economic and geo-climatic condition [5–9]. This necessitates a similar evaluation of the same in different geographical region of the country.

Here we determined the genotypes, prevalence and associated risk factors among women in Odisha with and without cervical cancer.

## Methods

### Study population and sample collection

Acharya Hari Hara Regional Cancer Center and SCB medical college, Cuttack, Odisha, the two apex referral hospitals of the state were considered to enroll subjects and cervical sample collection. This study was conducted between January 2014 and June 2016. Married women, above 18 years showing any of the symptoms like abnormal vaginal bleeding/discharge, pain during coitus, lower abdominal pain and clinician suspicion of cervical malignancy were included in the study after clinical examination by a gynecologist. Unmarried women, pregnant cases and patients undergoing treatment were excluded. Subjects were enrolled after getting informed written consent from them. This study is approved by the ethics committee of Regional medical research center (ICMR), Bhubaneswar, Odisha, India.

Clinical data including signs and symptoms and socio-demographic data such as age, education, economic status, age at marriage, the age of menopause were collected by interviewing the patients with a predesigned questionnaire.

Cervical swab specimen was collected using cytobrush and stored inside the viral transport media (Hi-Media) and transported to Virology laboratory, Regional medical research center, Bhubaneswar, Odisha for further analysis. Pap smears were prepared from the collected cervical sample for cytological analysis. Cytological classification was done according to Bethesda system [10]. Part of the tissue biopsy samples which was taken for diagnostic and patient management purpose were collected from confirmed cases of cervical carcinoma for histopathological analysis.

### DNA extraction and HPV DNA detection

DNA was extracted from a 200μl aliquot of exfoliated suspended cell samples using the QIA amp DNA Blood Mini Kit (Qiagen) as per the manufacturer’s instructions. Amplification of the human β-globin gene was performed to test sample sufficiency. To detect HPV, PCR was performed by using PGMY09/PGMY11 primers (MY11: 50-GCMCAGGGWCATAAYAATGG-30; MY09: 50-CGTCCMARRGGAWACTGATC-30–) targeting a 450-bp region of the HPV L1 gene.

### Genotyping by sequencing

L1 PCR products were isolated from agarose gel and purified. Sequencing was done using Big Dye Terminator sequencing kit (Applied Biosystems, Foster City, CA, USA) [11] and analyzed in silico on ABI 3100 Genetic Analyzer (Applied Biosystem). The results were then compared with the sequences available in the Genebank database using the National Center for Biotechnology Information BLAST program (http://www.ncbi.nlm.nih.gov/BLAST/).

### Genotyping of HPV by nested type specific multiplex E6/E7 PCR

PCR followed by sequencing didn’t resolve multiple infections. Hence, E6/E7 type-specific PCRs were performed to confirm multiple types. Type-specific primers were used to detect 18 HPV genotypes that include HPV16, HPV18, HPV31, HPV 33, HPV 35, HPV39, HPV 56, HPV 59, HPV 45, HPV 51, HPV 52, HPV 58, HPV 66, and HPV 68, HPV 6/11, HPV 42, HPV 43, and HPV 44 genotypes. Primers were obtained by following the available literature [12]. The whole process includes two PCR reaction; the first PCR using E6/E7 primers followed by the type-specific nested PCR.

Briefly,50 μl PCR reaction mixture containing, 10X Taq buffer with Mgcl2 (Biotools, Spain), 250mM dNTP (Biotools, Spain), 5U Taq DNA polymerase (Biotools, Spain), 20 pmol of each primer and 50ng of DNA was processed for the first PCR cycle. The cycling parameters were composed of 4 min of an initial denaturation step at 94 °C, followed by 35 amplification cycle. Each cycle includes the 30s of denaturation at 94 °C, 30s of annealing at 56 °C, elongation of 72 °C for 45s and a final extension of 4 min at 72 °C. Nested PCR reaction was same as the first PCR except for primers and sample. 2 μl of the PCR product was used for the nested PCRs. 10μl of the amplification products was analyzed by electrophoresis on 2% agarose gels stained with ethidium bromide followed by visualizing under UV light.

### Statistical analysis

Multivariate logistic regression analysis was performed to determine the risk factors associated with HPV infection. Bivariate analysis was done to determine the variables associated with multiple infections in cervical cancer cases. Statistical analyses were performed by using SPSS version 20.0 software and MediCal version 14.10.2 [13]. A *p*-value of ≤ 0.05 was considered as statistically significant.

Low and a high estimate of vaccine impact were Calculated by using the following formula:

For the quadrivalent vaccine, low estimate was the prevalence of HPV 6/11/16/18 genotypes alone or in association but excluding presence of another HPV type; high estimate was the prevalence of HPV 6/11/16/18 genotypes alone or in association possibly in presence of another HPV type. For the nonavalent vaccine, low estimate was the prevalence of HPV 6/11/16/18/31/33/45/52/58 genotypes alone or in association but excluding presence of another HPV type; high estimate was the prevalence of HPV 6/11/16/18/31/33/45/52/58 genotypes alone or in association possibly in presence of another HPV type [14]. To estimate the impact of respective vaccine, the average of the low and high estimate was considered.

Proportion of additional cases potentially prevented by one vaccine compared to the other vaccine was calculated as per the previously published study [14].

## Result

**Fig. 1:**
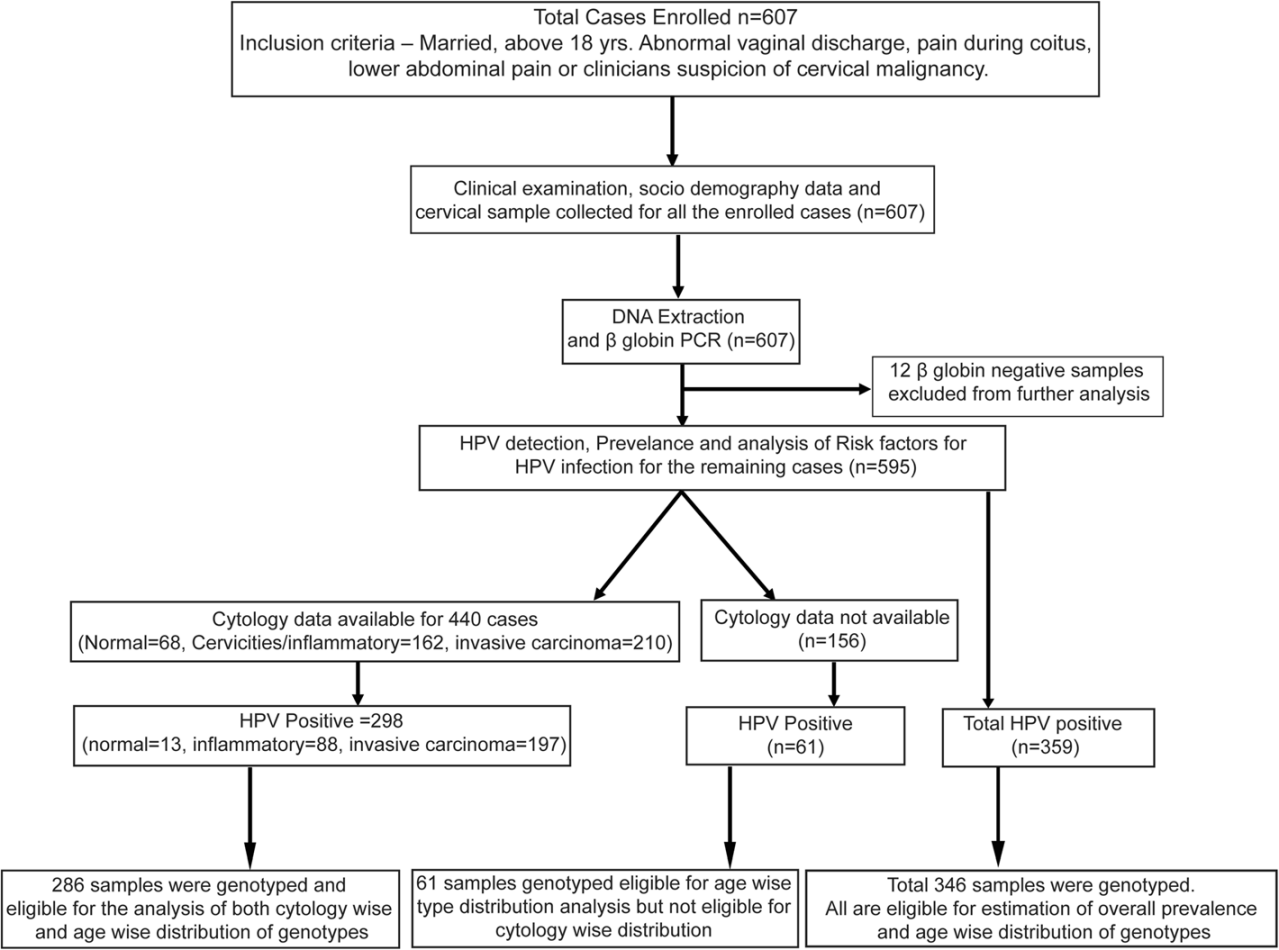
Enrollment of cases and outcomes

Enrollment of cases and outcomes were presented in Fig. 1. A total of 607 participants were being enrolled in the study. Among all the enrolled cases 12 cases were excluded from further analysis as they were negative for beta globin PCR. Cytological information was known for 440 cases only which includes normal (*n* = 68), cervicities (*n* = 162), invasive carcinoma (*n* = 210).

### Demographic and clinical features of cases

Socio-demographic features of 595 subjects are presented in Table 1. Mean and median age of the enrolled cases were 48.47(SD =12.47) and 48.5 years respectively with an age range of 19–86 years. The mean age of marriage was 19.97(SD = 3.98) years with an age range of 14–37 years. 401 cases were with parity ≤ 3 and 255 cases were in the postmenopausal state (Table 1). A majority of the cases (83.52%) were illiterate/just literate and belongs to low socio-economic class (60.33%).

**Table 1:** Sociodemographic and personal characteristics of the population(*n* = 595) as risk factors for HPV infection (*n* = 595)

Factors	HPV + ve *n* = 359	HPV –ve *n* = 236	OR (95% CI)	*P*
Age > 45 *N* = 369	236	133	1.4 (1.06-2.08)	.02
Age ≤ 45 *n* = 226	123	103		
Parity >3 *n* = 194	136	58	1.87 (1.29-2.69)	.0008
Parity ≤ 3 *n* = 401	223	178		
Contraceptive Yes *n* = 557	335	222	0.88 (.44-1.73)	.71
No *n* = 38	24	14		
Age of marriage ≤ 18 *n* = 234	153	81	1.42 (1.01- 1.99)	.043
Age of marriage > 18 *n* = 361	206	155		
Tobacco/betel Yes *n* = 289	179	110	1.13 (0.81- 1.58)	.43
No *n* = 306	180	126		
Education No *n* = 497	304	193	1.18 (0.76- 1.83)	0.4567
Yes *n* = 98	56	42		
Low socioeconomic condition *n* = 359	245	114	1.62 (1.13- 2.31)	0.0081
High socioeconomic condition *n* = 200	114	86		
Rural *n* = 361	322	39	20.97 (12.65-34.76)	<0.0001
Urban *n* = 131	37	94		
Poor Menstrual hygiene *n* = 492	306	186	1.55 (1.01-2..37)	0.0437
Good Menstrual hygiene *n* = 103	53	50		
Post menopause *n* = 255	170	97	1.85 (1.33 to 2.57)	0.0002
Pre menopause *n* = 340	165	175		

The common clinical features recorded were abnormal discharge with or without blood stain (55%), postmenopausal bleeding (20%), bleeding and pain during coitus (2%), lower abdominal pain (17%), intermenstrual bleeding (14%), prolapse (6%), and swelling abdomen (1%).

### Clinicopathological characteristics of ICC cases

As per the International Federation of Gynecology and Obstetrics (FIGO), ICC cases were classified into different stages which include IB (*n* = 9), IIA (*n* = 8), IIB (=38), IIIB (*n* = 111), IIIA (*n* = 2) and IVA (*n* = 4). The histopathological result of 172 cases showed that 167 cases belonged to Squamous cell carcinoma (SCC) and only 5 cases identified with Adenocarcinoma (ADC). Based on the available data for 89 cases, only 30 cases had tumor size ≥2 cm and 59 cases had <2cm. Similarly, out of 86 available reports 30 cases had lymph node metastasis whereas 56 cases didn’t have lymph node metastasis (Additional file 1: Table S5).

### Prevalence and analysis of risk factors for HPV infection

The overall prevalence of HPV infection was found to be (359/595) 60.33%. Prevalence of HPV infection was 93.80% (197/210) in invasive cervical cancer (ICC) cases, 54.3% (88/162) in inflammatory smear and 19.11% (13/68) in normal cases (Fig. 1). Risk factors for HPV infection were analyzed by multivariate logistic regression analysis (Table 1). Age group > 45 years, parity ≥3, low socio-economic condition, rural residential and postmenopausal states were found to be significantly associated with HPV infection.

### HPV genotype distribution

Three hundred forty-six samples were processed for genotyping by type-specific nested multiplex PCR. The most commonly detected genotype was HPV16 (87.28%) followed by HPV18 (24.56%) (Additional file 1: Table S1). Other detected genotypes in descending order were HPV 51(3.46%), HPV 39(3.17%), HPV 66(2.8%), HPV 68(2.3%), HPV 35(1.7%), HPV 45(1.7%), HPV 44(1.1%), HPV 58 (1.1%), HPV 52(.57%), HPV 6/11(.57%), HPV 42(1.1%) and HPV 43(.57%) (Additional file 1: Table S1). Prevalence of single and multiple genotypes was 76.58% and 23.41% respectively (Additional file 1: Table S1).

Cytology-wise HPV genotype distribution was done in 286 samples which includes normal cytology (*n* = 13), inflammatory smear (*n* = 88) and ICC (*n* = 185) (Additional file 1: Table S2). Among the women with normal cytology, 53.84% were infected with single genotype of HPV16 while rests were infected with coinfection of HPV16 and 18. No other genotypes were observed in this group (Additional file 1: Table S2).

Among the women with inflammatory cytology HPV 16(89.77%) was the most predominant genotype followed by HPV 18(28.4%). Other genotypes detected among this group were HPV 66(6.8%), HPV 68(6.8%), HPV51 (3.4%) and HPV35 (2.27%) (Additional file 1: Table S3). In this group 70.45% cases were infected with single genotypes while 29.54% cases were infected with multiple HPV types (Additional file 1: Table S2). Infections with double genotypes found in 19.31% of cases while 10.22% cases were infected with triple genotypes combinations (Additional file 1: Table S2). The most common combination of coinfection in this group was HPV 16 + 18 (14.72%) followed by HPV16 + 66 + 68(6.81%) (Additional file 1: Table S2).

Among invasive cancer cases the most prevalent genotype was HPV16 (83.78%) followed by HPV 18(21.08%) and HPV 51(5.4%) (Additional file 1: Table S3). Other genotypes in invasive cancer were HPV 45, HPV35, HPV66, HPV68, HPV44, HPV43, HPV42, HPV58 and HPV 52(Additional file 1: Table S3). Among the ICC cases78.9% cases were infected with single genotypes while 21.08% cases were with multiple genotypes including double (11.89%), triple (5.94%) and quadruple (3.24%) combinations of genotypes (Additional file 1: Table S2). The most prevalent genotype combination as coinfection among ICC cases was HPV16 + 18(4.32%) followed by HPV16 + 39(2.16%) (Additional file 1: Table S2).

Single genotype increases with the severity of the lesion. Single HPV16 also increases with the disease severity and highest in cervical carcinoma cases (Additional file 1: Table S2). Prevalence of HPV16 + 18 decreases with lesion severity and found to be lowest in ICC cases (Additional file 1: Table S2). The number of genotypes increases with the grade of the lesion (Additional file 1: Table S2).

Distribution of high-risk and low-risk HPV genotypes in the study population has been summarized in Additional file 1: Table S3. No low-risk genotypes were detected among the cases with normal and inflammatory cytology. All the cases with inflammatory cytology were infected with high risk genotypes except 6 cases which were infected with HPV66, an intermediate risk genotype. High risk genotypes were detected in all the ICC cases. Besides, low-risk (6.4%) and intermediate-risk genotype (2.16%) were also detected in ICC cases but in association with the high-risk genotypes. The low risk genotypes detected were HPV6/11, HPV44, HPV43 and HPV42 while HPV 66 was the only intermediate risk genotype.

### Prevalence and distribution of HPV genotypes infection in different age group

**Fig. 2:**
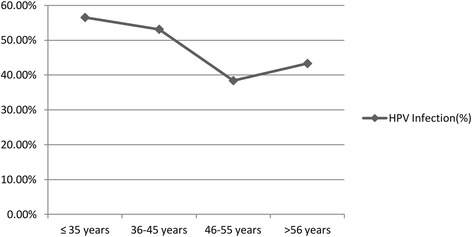
Age wise prevalence of HPV infection in women without cancer (inflammatory and normal cytology)

**Fig. 3:**
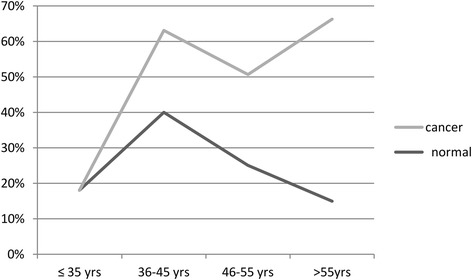
Trends of Multiple infections among different age group in normal and cervical cancer

Irrespective of cytology, the overall prevalence of HPV infection was highest (71.25%) among women aged >55 years (Additional file 1: Table S4). The age wise HPV infection curve for the cases without cancer depicted a bimodal peak as shown in Fig. 2. It shows two peaks, one at the age of ≤ 35 years with highest prevalence and another at > 55 years. Figure 3 shows the age wise prevalence of multiple infections in women with and without cancer. Among the women without cancer the prevalence of multiple genotypes was highest in women aged between 36 and 45 years while in ICC cases it is highest among women aged >55 years. Among ICC cases the curve was bimodal showing two peaks, one at the age of 36–45 years and another at the age of > 55 years. But in normal cases only one peak at the age of 36–45 years was observed.

### Impact of 2v, 4v and 9v vaccine in preventing cervical carcinoma in Odisha

Low estimates of genotypes targeted by 2v, 4v, and 9v vaccine were 152(82.16%), 152(82.6%) and156 (84.32%) respectively. High estimates of genotypes targeted by 2v, 4v, and 9v vaccines were 181(97.83%), 183(98.91%) and 185(100%) respectively. Absolute impact of 2v, 4v and 9v were 89.99, 91.65 and 92.16% respectively. The additional impact of 9v was increased when compared with 4v and 2v (Table 2).

**Table 2:** Impact of 2v, 4v and 9v vaccine based on the ICC cases infected with vaccine targeted genotype

	Lowest estimation	Highest estimation	(LE + HE/2)%
Cases infected with genotypes targeted by 2v vaccine(HPV16/18)	152 (82.16%)	181 (97.83%)	89.99%
Cases infected with genotypes targeted by 4v vaccine(HPV16/18/6/11)	152 (82.6%)	183 (98.91%)	91.65%
Cases infected with genotypes targeted by 9v vaccine(HPV16/18/6/11/31/33/45/52/58)	156 (84.32%)	185 (100%)	92.16%
Absolute additional impact of 4v vaccine (compared with 2v vaccine)	-	1.08%	1.08%
Relative additional impact of 4v vaccine (compared with 2v vaccine)	-	1.09%	1.09%
Absolute additional impact of 9v vaccine(compared with 2v vaccine)	2.1%	2.16%	2.16%
Relative additional impact of 9v (compared with 2v vaccine)	2.4%	2.16%	2.28%
Absolute additional impact of 9v vaccine (compared with 4v vaccine)	2.1%	2.16%	2.16%
Relative additional impact of 9v vaccine (compared with 4v vaccine)	1.08%	1.09%	1.085%

*LE* Low estimate, *HE* High estimate

### Association between clinicopathological variables with multiple genotypes

Association of clinicopathological variables with multiple genotypes have been shown in Additional file 1: Table S5. Among the ICC cases, the proportion of multiple types was more common in patients with aged > 50 years as compared with aged ≤ 50 years. The frequency of multiple genotypes was higher among patients diagnosed at a late stage than those at an early stage. Frequency of multiple infections was also higher in the cases with lymphnode metastasis than the cases without LN metastasis. No significant association was found between multiple genotypes and clinicopathological variables such as age at diagnosis (≤50 years *vs* >50 years), stage at diagnosis (early stage or stage I-II *vs* late stage or stage III-IV), lymph node metastasis (yes *vs* no) and histopathology (Squamous cell carcinoma *vs* Adenocarcinoma). Multiple types were found to be significantly associated with tumor size ≥ 2cm (OR = 4.95 95% CI = 1.68–14.51 *p* = .0035) (Additional file 1: Table S5).

## Discussion

The prevalence of cancer cervix is high in India [1] but countrywide data on HPV infection and genotype distribution is not available which would have been useful for a wider vaccination program. To the best of our knowledge, the current study is the first to report the prevalence of HPV infection and genotype distribution among the women with cervical diseases in the state of Odisha. Odisha is an eastern state of the Indian subcontinent covering an area of 1155,820 km with 45 million populations, known for her socio-economic backwardness and various public health issues. Samples were obtained from two apex referral hospital to which patients from all the districts of Odisha visit for consultation. Therefore, the study provides a precise estimation of the HPV prevalence and genotype distribution in symptomatic women of the state.

Compared to other studies in India, the present study disclosed a high prevalence of HPV infection among the women with normal cytology showing minor gynecological complaint [5, 6, 15–17]. This study, however, did not look for any bacterial, fungal or HIV infection which could make them prone to HPV infection in symptomatic cases.

Prevalence of HPV among the cervical cancer cases was 94.28% in the present study. It is generally accepted that HPV virtually causes 100% of cervical carcinoma. Hence, the difference in results could be partly explained by differences in the sensitivity of the HPV detection techniques used. The most prevalent genotype in cervical cancer was HPV 16 followed by18 is in accordance with international and local data [7, 8, 16]. As compared to other studies, the overall prevalence of HPV 16 and 18 in cancer cases (82.3%) is much higher in the present work [7, 8, 16].

Analysis of genotypes distribution in ICC cases showed that HPV 16(83.78%) and 18(21.08%) were the most predominant genotypes which is quite similar to the studies reported from India and worldwide. In Kolkata, 59–74% of ICC cases are infected with HPV 16 and 2–13.9% cases infected with HPV 18 [7, 18]. Reports from south India showed HPV 16 and HPV18 accounts for 58–69% and 5–19.4% of ICC cases respectively [16, 18]. In a similar study from Delhi (north India) reported that HPV 16 and 18 contributing 73.6 and 14.2% cases of cervical carcinoma [19]. Other parts of Central and west India also have similar reports showing HPV 16(72–73.6%) as the most predominant genotype followed by HPV18 (5–11.9%) in cervical carcinoma cases [18, 20]. In Pakistan, HPV 16 and 18 accounts for 45.1–94.9% and 1.7–43.1% of ICC cases respectively [21–24]. However, in the Chinese population, HPV 16 is the most predominant followed by HPV 52 rather than 18 [25]. Distribution of most common genotypes in ICC cases is also consistent with the types found in worldwide [26, 27].

HPV 51 was found to be the 3rd most predominant genotype in the present study, though it is rarely reported in other regions of the country and worldwide. Data on the prevalence and distribution of three most prominent genotypes in the cancer cases from different geographical regions of India shows a great regional variation [5–9, 15–17].

HPV 66, an intermediate risk genotype, had a prevalence of about 3% and a predominant type in cervicitis cases in the present study is rare in other population. The presence of HPV66 across all age groups and predominantly in cervicitis might indicate its possible role in the development of cervical cancer by triggering inflammation and persistent infection.

Low-risk HPV infection is a rare event in this population. Low-risk infection was observed in ICC cases only and always present in association with the high-risk genotypes. It remains unclear whether the association of low-risk genotypes with the high risk induces the progression of lesion or is it the effect of high-risk HPV that makes prone to the infection of other types.

The present study showed highest infection rate at the age of ≤35 years among women without cancer. The age-specific trend of HPV infection among noncancer cases showed bimodal shaped infection peak which is similar to many other reports [28]. HPV infection at younger age reached its peak soon after sexual initiation [28].

Age specific multiple HPV genotype infection curve for ICC cases shows a bimodal peak. Highest peak is at > 55 years and another shift is at 36–45 years (Fig. 3). In women without cancer multiple infections were most frequently observed in the age group 36–45 years and lowest in >55 years age group which corroborates with previous studies [25, 29]. Since sexual activity is higher in younger women, multiple genotype infection is higher at this age [30–32]. The higher frequency of multiple infections at the 36–45 years in both cancer and non cancer group might be due to multiple sexual partners at this age. The frequency of HPV infection and multiple types increases with age explains cumulative lifetime exposure [33], relative incompetency in viral clearance and insufficient adaptive immune responses at this age caused by hormonal changes at menopausal transition, contributing to HPV persistence or reactivation of latent HPV infections [34, 35].

The present study showed a significant association of multiple infections with increased tumor size. However, no significant association of multiple infections with other clinicopathological variables (age, stage, lymph node metastasis and histopathology) was found in the present work which agrees with the findings of Mungala et al [36]. Prognosis of cervical carcinoma is related to clinical stage, lymph node metastasis, parametrial invasion, primary tumor size, histological type, depth of cervical stromal invasion and lymph vascular space involvement [37]. Bachtiary et al. [38], in a study of 106 cervical cancer patients receiving radiation therapy, found that multiple-type HPV was an independent poor prognostic factor. Though there are several reports showing the association of multiple infections with cervical cancer [39, 40] the biological effect of multiple infections on cervical diseases has not yet been established. Synergistic effect of multiple infections by HPV genotypes on carcinogenesis is supported by many studies [7–9, 17, 26], while contradicting studies [25, 29, 31, 32, 35] invoke confusion. Association of multiple infections with tumor size is an interesting finding in this regard. Analysis on the correlation of tumor size with lymph node metastasis is under consideration which would throw more light on this.

The rate of single genotype infection especially HPV 16, was very high in the abnormal histopathological group as compared to the normal group which is in accordance with Baloch et al [28]. Hence, even without the information of the other genotypes, attention should be paid to those infected with single HPV16. Cervical cancer screening could be improved by detection of HPV16 besides cytology.

People staying in a rural area had 20-fold higher risk of acquiring HPV infection. Rural residential area reflects poor socio-economic condition possibly related to lack of access to proper care which facilitates infection and persistence of HPV and an increasing risk of cancer development [41]. In rural Odisha where sexual conception and behavior of women are conservative, men possessing multiple sexual partners might be a major source of HPV infection in a female. People from rural Odisha goes to the cities of neighboring states for their livelihood which provides an opportunity for having multiple partners. Our finding supports close surveillance of elderly women with HR-HPV infection and women of the rural area.

Approximately, more than 70% worldwide ICC could be prevented by the available bivalent HPV vaccines-CervarixTM (GlaxoSmithKline Biologicals, Rixensart, Belgium) and quadrivalent HPV vaccine- Gardasil® (4vHPV) (Merck & Co., Inc., Kenilworth, NJ, USA) [26, 27]. The recently approved 9-valent vaccine covers HPV16/18/31/33/45/52/58 genotypes [42, 43].

**Fig. 4:**
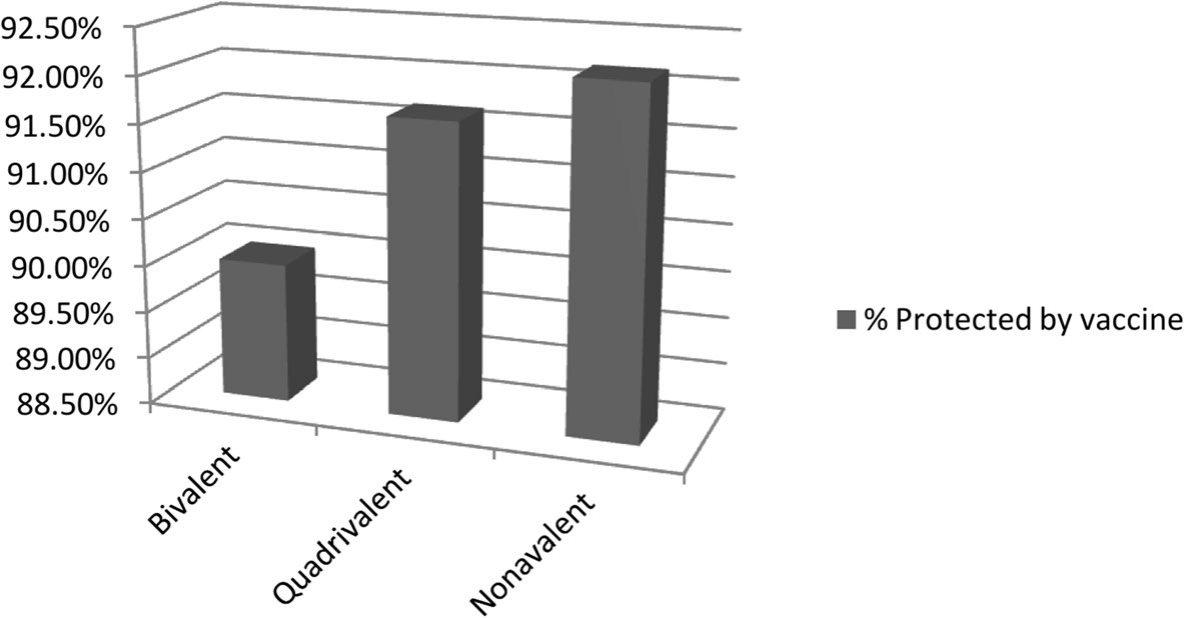
Potential Impact of different HPV vaccines to prevent cervical cancer in Odisha

To know the impact of a vaccine, estimating the attribution of cases to HPV types is confusing due to multiple genotypes present in the same tumor. In the present study high and low estimate of the impact of vaccines has been estimated to overcome this difficulty. Since the true potential impact lies somewhere between the high and low estimate [14] the average value of the two was considered for convenience. The present study shows a potential vaccine impact of 2v and 4v vaccines were 89.99 and 91.65% respectively. The impact would increase up to 92% with introducing 9v vaccine (Fig 4). It is similar to a study in French population [14]. The potential impact of the nonavalent vaccine in Asia and worldwide are 91.5 and 89.5% respectively [44]. Inconsistent with other studies, our findings also show an increase in the efficacy of vaccine with the switch from bivalent or tetravalent to the nonavalent vaccine [14, 45]. The result shows, for a maximum effectiveness of vaccination program, a 9v vaccine would be more useful.

HPV51, the third most prevalent genotype, and HPV66 another more frequent genotype of this region can’t be cross protected even by the new 9v vaccine for being genetically unrelated. The risk of development of invasive cervical carcinoma associated with these genotypes needs to be estimated.

We were unable to assess HIV status or another disease for our participants, which could strongly influence the prevalence of HPV infection.

## Conclusions

The present study provides first information about the genotype distribution among women with cervical cancer and without cancer cases of the state of Odisha which would help planning an appropriate strategy for disease monitoring. This study also offers the baseline data for future research and post-vaccination surveillance in the region. The nonavalent vaccine would be the most effective vaccine to prevent cervical carcinoma in Odisha. Hence an effective vaccination program based on regional HPV epidemiological profile along with the cervical cancer screening is necessary to reduce the cervical cancer burden in India. Besides, HPV surveillance targeting elderly women and rural people will be useful.

## Additional file

Additional file 1: Table S1. Distribution of HPV genotypes (*n* = 346). **Table S3.** Distribution of Low risk and high risk genotypes in different cytology. **Table S4.** HPV infection in different age group. **Table S5.** Clinico-Pathological characteristics in ICC patients and their association with multiple genotypes. (DOCX 22 kb)
