# Supplementary material for: HPV Genotypes distribution in Indian women with and without cervical carcinoma: Implication for HPV vaccination program in Odisha, Eastern India
Source: BMC Infect Dis. 2017 Jan 5;17:30. doi: 10.1186/s12879-016-2136-4 (PMC5216564; doi:10.1186/s12879-016-2136-4)
Supplement: Additional file 1: Table S1. — Distribution of HPV genotypes (n = 346). Table S3. Distribution of Low risk and high risk genotypes in different cytology. Table S4. HPV infection in different age group. Table S5. Clinico-Pathological characteristics in ICC patients and their association with multiple genotypes. (DOCX 22 kb) [file 12879_2016_2136_MOESM1_ESM.docx]

**SUPPLIMENTARY MATERIALS**

**Table S1 : Distribution of HPV genotypes (n=346)**

| Genotypes | Prevalence of HPV genotype (%) |
| --- | --- |
| Single genotype | 265(76.58) |
| Multiple genotype | 81(23.41) |
| HPV 16 | 302(87.28) |
| HPV18 | 85(24.56) |
| HPV51 | 12(3.46) |
| HPV39 | 11(3.17) |
| HPV45 | 6(1.7) |
| HPV66 | 10(2.89) |
| HPV68 | 10(2.89) |
| HPV35 | 6(1.7) |
| HPV58 | 4(1.15) |
| HPV42 | 4(1.15) |
| HPV43 | 2(.57) |
| HPV6 | 2(.57) |
| HPV11 | 2(.57) |
| HPV52 | 2(.57) |
| HPV44 | 4(1.15) |

**TABLE S2 Genotypes distribution in different cyto-pathological conditions**

| **Genotype** | **Total**  **N=286** | **Normal**  **N=13** | **Inflammatory**  **N=88** | **Invasive cancer**  **N=185** |
| --- | --- | --- | --- | --- |
| Single | 215(75.17%) | 7(53.84%) | 62(70.45%) | 146(78.9%) |
| Multiple | 71(24.82%) | 6(46.15%) | 26(29.54%) | 39(21.087%) |
| **Single genotypes** |  |  |  |  |
| HPV16 | 187(65.35%) | 7(53.84%) | 55(62.5%) | 125(67.56%) |
| HPV18 | 26(9.09%) | 0 | 7(7.9%) | 19(10.27%) |
| HPV45 | 2(.69%) | 0 | 0 | 2(1.08%) |
| **Combination in double infection** | 45(15.73%) | 6(46.15%) | 17(19.31%) | 22(11.89%) |
| HPV16+18 | 27(9.4%) | 6(46.15%) | 13(14.72%) | 8(4.32%) |
| HPV16+44 | 2(.69%) | 0 | 0 | 2(1.08%) |
| HPV16+35 | 4(1.3%) | 0 | 2(2.27%) | 2(1.08%) |
| HPV16+39 | 4(1.3%) | 0 | 0 | 4(2.16%) |
| HPV16+51 | 1(.34%) | 0 | 0 | 1(.54%) |
| HPV18+39 | 3(1.04%) | 0 | 0 | 3(1.62%) |
| HPV18+45 | 2(.69%) | 0 | 2(2.27%) | 0 |
| HPV18+51 | 2(.69%) | 0 | 0 | 2(1.08%) |
| **Combination in triple infection** | 20(6.9%) | 0 | 9(10.22%) | 11(5.94%) |
| HPV16+66+68 | 8(2.7%) | 0 | 6(6.81%) | 2(1.08%) |
| HPV16+66+51 | 2(.69%) | 0 | 0 | 2(1.08%) |
| HPV16+18+39 | 3(1.04%) | 0 | 0 | 3(1.62%) |
| HPV16+18+58 | 2(.69%) | 0 | 0 | 2(1.08%) |
| HPV16+18+51 | 3(1.04%) | 0 | 3(3.4%) | 0 |
| HPV 58+35+42 | 2(.69%) | 0 | 0 | 2(1.08%) |
| **Combinations in quadruple infection** | 6(2.09%) | 0 | 0 | 6(3.24%) |
| HPV16+51+52+42 | 2(.69%) | 0 | 0 | 2(1.08%) |
| HPV 16+6/11+43 | 2(.69%) | 0 | 0 | 2(1.08%) |
| HPV18+45+44+51 | 2(.69%) | 0 | 0 | 2(1.08%) |

**Table S3- Distribution of Low risk and high risk genotypes in different cytology**

| **Genotypes** | **Normal n=13 (%)** | **Inflammatory n= 88 (%)** | **Invasive n=185 (%)** |
| --- | --- | --- | --- |
| High risk HPV | All | All | All |
| HPV 16 | All | 79(89.77) | 155(83.78) |
| HPV 18 | 6(46.15) | 25(28.4) | 39(21.08) |
| HPV 35 | 0 | 2(2.27) | 4(1.08) |
| HPV 39 | 0 | 0 | 10(5.4) |
| HPV 51 | 0 | 3(3.4) | 9(4.8) |
| HPV 52 | 0 | 0 | 2(1.08) |
| HPV 68 | 0 | 6 | 2(1.08) |
| HPV 45 | 0 | 2 | 4(2.16) |
| HPV 58 | 0 | 0 | 4(2.16) |
| Low risk HPV | 0 | 0 | 12(6.4) |
| HPV6/11 | 0 | 0 | 2(1.08) |
| HPV44 | 0 | 0 | 4(2.16) |
| HPV43 | 0 | 0 | 2(1.08) |
| HPV 42 | 0 | 0 | 4(2.16) |
| Intermediate risk | 0 | 6(6.8) | 4(2.16) |
| HPV 66 | 0 | 6(6.8) | 4(2.16) |

**Table S4 HPV infection in different age group**

| Age(yrs) | HPV infection (%) |
| --- | --- |
| ≤ 35 yrs | 53 |
| 36-45 yrs | 57.4 |
| 46-55 yrs | 57.4 |
| >55 | 71.25 |

**TABLE S5 Clinico-Pathological characteristics in ICC patients and their association with multiple genotypes**

| Clinic-pathological variables | Multiple | Single | Odds ratio | *P* |
| --- | --- | --- | --- | --- |
| Age |  |  |  |  |
| Below equal 50 n=64 | 13(20.31%) | 51(79.68%) | 1.24(0.58 to 2.63) | .05 |
| Above 50 n=121 | 26(21.48%) | 95(78.51%) |  |  |
| FIGO STAGE |  |  |  |  |
| Late stage (III-IV) n=117 | 28(23%) | 89(76.06%) | 0.79(0.36 to 1.74) | .56 |
| Early stage(I-II) n=55 | 11(20%) | 44(80%) |  |  |
| TUMOR SIZE |  |  |  |  |
| ≥2cm n=30 | 12(40%) | 18(60%) | 4.95(1.68 to 14.51) | .0035 |
| <2cm n=59 | 7(11.86%) | 52(88.13%) |  |  |
| Lymphnode metastasis |  |  |  |  |
| Yes  N=30 | 15(50%) | 15(50%) | 2.11(0.85 to 5.24) | .107 |
| No n=56 | 18(31%) | 38(69%) |  |  |
| CELL TYPE |  |  |  |  |
| Squamous cell carcinoma | 30(17.96%) | 137(82.03%) | 0.328(0.05 to 2.05) | 0.23 |
| Adenocarcinoma n=5 | 2(40%) | 3(60%) |  |  |
